# Annotations

**Published:** 1886-10-16

**Authors:** 


### Annotations.

Dangerous Reticence.

We are informed that the following inquiries were addressed to the principal charities throughout the metropolis, with the view of ascertaining how far the prevailing depression had affected their voluntary income:—"Please state on enclosed form the amounts received in each of the years 1876, 1880, 1885, from (1) Subscriptions and Donations; (2) Church Collections; (3) Legacies; (4) Other Voluntary Sources." Prompt and precise answers were received from all except one institution, the Earlswood Asylum for Idiots, the secretary of which wrote: "I have failed in obtaining permission to send you the details you ask for." We have reason to believe that it is the practice of the managers of the Earlswood Asylum, although it is supported mainly by voluntary contributions, to refuse to give information concerning its management and work. Those who are best able to form a judgment in such matters contrast this institution with the Royal Albert Asylum, Lancaster, which is also supported by voluntary contributions, and from which the fullest information is at all times obtainable on inquiry. It is not our purpose to-day to compare the management of these two institutions in other respects, or it might not be difficult to show, that reticence in supplying information for public purposes, is generally found connected with relative inefficiency in the administration of public institutions. Apart from other considerations, it is certain that those who give most to charities are becoming more alive to the importance of ascertaining in the first instance how they are managed. It follows, therefore, that an institution like the Earlswood Asylum, which makes it an habitual practice to decline to give reasonable information in response to courteous inquiry, must suffer, and, as we think, deservedly suffer, pecuniary loss in consequence.

Hospitals on the Rates.

Lord Derby, in the terse and able address he has sent us, which we publish in another column, deprecates the maintenance of hospitals by public aid, in the shape of grants, either by local rates or general taxation. Assistance from the rates, if not vicious in principle, because a hospital has certainly as large a claim on the score of public utility as a school for drawing, would still be decidedly objectionable. The support of hospitals, rightly understood, is not only the duty, but the privilege of all classes. No thoughtful person will say that every one who gets ill has a right to physic, or that A., who is industrious and lays up for a rainy day, and so pays for his own physic, is bound to pay for B. who drinks away his money and so cannot do likewise. We would urge, with Mr. Haweis, "Don't say the State ought to provide hospitals, and the solvent taxpayer to be mulcted regularly for the public sick-house, as he is for the public poor-house. All hopeful economists and all wise philosophers look forward to the abolition of workhouses, and to the re-introduction, as in Germany, of the voluntary system in some form. The Government, as Mr. Herbert Spencer has so well pointed out, may do too much, as well as too little, for the individual. To give everyone a legal right to maintenance, to physic, to clothes, or to anything else at the public expense, is to encourage improvidence and to check thrift. All hospital or poor relief, to be sound, should be on the voluntary principle ; and all voluntary aid, to be sound, should be on the definite understanding that in such a world as ours, however dangerous it may be to do for people what they ought to do for themselves, there must arise cases of emergency, exceptions to the rule, which can be met by nothing but public and private benevolence." Depend upon it, Mr. Haweis is right, and it would be a bad day for England, and especially for her people, if all the hospitals were paid for out of the rates. Let our readers remember that the Eastern Hospital, with its scandalous abuses, so recently made public, is a rate-supported hospital. Such scandals are the product of rate-supported institutions, but the voluntary system has produced efficiency almost everywhere. This is a working man's question, and of all classes it is to his interest to prevent the voluntary hospitals from going on the rates.

The New Order of Things.

One result of the new order of things has been to produce new sources of income for the hospitals. Previous to 1870, with the exception, perhaps, of the contribution of the magnificent sum of £6,000 per annum to the Glasgow Royal Infirmary by the artisans on the Clyde, hospitals received little pecuniary assistance from the working classes. A movement was set on foot by the late Mr. Sampson Gamgee at Birmingham in 1870, which resulted in a free contribution from the working classes of upwards of £4,000 towards the extension fund of the Queen's Hospital. In this case the invariable rule applied, and once having tasted the pleasures of giving, the working men of Birmingham determined to nurture, extend, and develop the source of pleasure they had thus discovered by instituting Hospital Saturday. Tentatively, but unsatisfactorily, others had attempted or discussed the possibility of grafting Hospital Saturday collections upon the Hospital Sunday movement. It was left to the men of Birmingham, however, with whose indomitable energy and commendable public spirit the country has long been familiar, to make Hospital Saturday not only a success, but worthy in every way of the working classes. In six weeks, and on the first Hospital Saturday, in the year 1873, they raised upwards of £5,000, which was presented as a free gift to the Birmingham Hospitals. No expenses were deducted from the collections, because this enthusiasm for the hospitals on the part of the working classes excited the sympathy of Mr. G. F. Muntz, who asked to be allowed to pay the whole expenditure himself. Thus a good deed is never lost, and he who plants kindness gathers love. A new departure has resulted from this action of the working men of Birmingham on behalf of the hospitals. Everywhere the artisans have shown a commendable determination to take their part in the privilege of contributing something to relieve the sick and suffering members of our race. The latest instance is to be found at Burnley, which has a population of less than 60,000, where the working classes have subscribed £5,000, to which the other residents have added £15,000 more, and so one of the most complete hospitals in the country has been successfully built.

"Hope for the Hopeless."

Under this heading *The Daily News* publishes a letter showing how a patient, presumably consumptive, who suffered from severe attacks of blood-spitting, was cured, as he himself believed, by inhaling the *Bacterium Termo* according to the method propounded by Dr. Cantani. It may be questioned whether, in the present state of knowledge on the subject, the publication of such a letter is either wise or kind. Persons who are suffering from phthisis at once think that if they could be placed under Cantani's treatment they would get well forthwith. Unhappily, the results of experiments, skilfully and conscientiously carried out according to Cantani's methods by experts in this country, do not give any support to his views, and the question of the value of his treatment must be regarded as still *sub judice.* With regard to the case in *The Daily News*, whilst it need not be doubted that the patient was the subject of severe hæmorrhages, or that he recovered after the inhalation of *Bacterium Termo*, it by no means follows that the inhalation was *the cause* of the cure. Every medical man of moderate experience can tell of a goodly number of such cases which have occurred in his own practice, and have recovered under various ordinary methods of treatment; some, indeed, without any treatment at all, other than quiet and rest. By all means let Cantani's or any other treatment which offers even a remote prospect of the cure of this dreadful disease, be made the subject of the most thorough and frequent experiment, until its value or its worthlessness is fully demonstrated. But in the meantime let us not raise hopes in the minds of the sick and their friends, which may be the cause of much cost, much suffering, and much grievous disappointment.

